# 1*H*-Benzimidazole-2(3*H*)-thione

**DOI:** 10.1107/S1600536809008058

**Published:** 2009-03-14

**Authors:** De-Cai Wang, Shan Mi, Wei Xu, Liang Jiang, Xin-Ming Huang

**Affiliations:** a State Key Laboratory of Materials-Oriented Chemical Engineering, College of Life Science and Pharmaceutical Engineering, Nanjing University of Technology, Xinmofan Road No. 5 Nanjing, Nanjing 210009, People’s Republic of China; b College of Science, Nanjing University of Technology, Xinmofan Road No. 5 Nanjing, Nanjing 210009, People’s Republic of China

## Abstract

The asymmetric unit of the title compound, C_7_H_6_N_2_S, contains one half-mol­ecule; the C and S atoms of the C=S group lie on a crystallographic mirror plane. In the crystal structure, inter­molecular N—H⋯S hydrogen bonds link the mol­ecules.

## Related literature

For a related structure, see: Mavrova *et al.* (2007 ▶). For bond-length data, see: Allen *et al.* (1987 ▶).
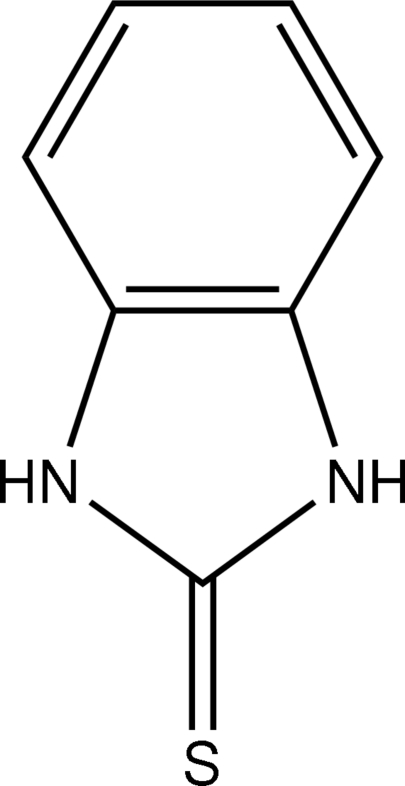


## Experimental

### 

#### Crystal data

C_7_H_6_N_2_S
*M* *_r_* = 150.21
Monoclinic, 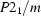

*a* = 4.915 (1) Å
*b* = 8.5590 (17) Å
*c* = 8.2920 (17) Å
β = 91.76 (3)°
*V* = 348.66 (12) Å^3^
*Z* = 2
Mo *K*α radiation
μ = 0.38 mm^−1^
*T* = 294 K
0.30 × 0.20 × 0.10 mm

#### Data collection

Enraf–Nonius CAD-4 diffractometer
Absorption correction: ψ scan (North *et al.*, 1968 ▶) *T*_min_ = 0.896, *T*_max_ = 0.963
903 measured reflections
813 independent reflections
647 reflections with *I* > 2σ(*I*)
*R*_int_ = 0.044
3 standard reflections frequency: 120 min intensity decay: 1%

#### Refinement

*R*[*F* ^2^ > 2σ(*F* ^2^)] = 0.049
*wR*(*F* ^2^) = 0.152
*S* = 1.00
813 reflections
45 parameters
H-atom parameters constrained
Δρ_max_ = 0.37 e Å^−3^
Δρ_min_ = −0.26 e Å^−3^

### 

Data collection: *CAD-4 Software* (Enraf–Nonius, 1989 ▶); cell refinement: *CAD-4 Software*; data reduction: *XCAD4* (Harms & Wocadlo, 1995 ▶); program(s) used to solve structure: *SHELXS97* (Sheldrick, 2008 ▶); program(s) used to refine structure: *SHELXL97* (Sheldrick, 2008 ▶); molecular graphics: *ORTEP-3 for Windows* (Farrugia, 1997 ▶) and *PLATON* (Spek, 2009); software used to prepare material for publication: *SHELXTL* (Sheldrick, 2008 ▶).

## Supplementary Material

Crystal structure: contains datablocks global, I. DOI: 10.1107/S1600536809008058/hk2638sup1.cif

Structure factors: contains datablocks I. DOI: 10.1107/S1600536809008058/hk2638Isup2.hkl

Additional supplementary materials:  crystallographic information; 3D view; checkCIF report

## Figures and Tables

**Table 1:** Hydrogen-bond geometry (Å, °)

*D*—H⋯*A*	*D*—H	H⋯*A*	*D*⋯*A*	*D*—H⋯*A*
N—H0*A*⋯S^i^	0.86	2.57	3.3798 (19)	158

Symmetry code: (i) 
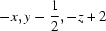
.

**Fig. 1.**
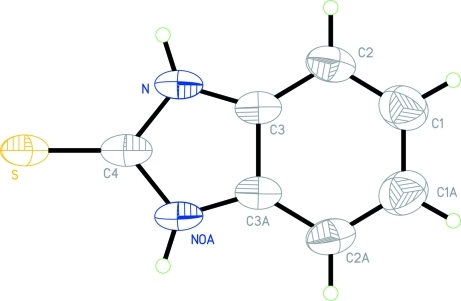
The molecular structure of the title molecule, with the atom-numbering scheme. Displacement ellipsoids are drawn at the 30% probability level.

**Fig. 2.**
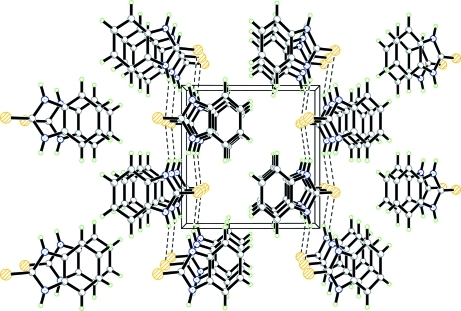
A partial packing diagram. Hydrogen bonds are shown as dashed lines.

## supplementary crystallographic information

### Comment

It is a kind of secondary age inhibitor, and could reinforce the effect combined with DNP AP and other nonpolluting age inhibitors. It disperses easily in rubber, and the color does not change under sun exposure. Its pollution capacity is limited. 2-Mercaptobenzimidiazole is a new kind of anti-leprosy drugs, and its toxicity is lower than sulphone drugs. It should not be used in the patients to which can not be given sulphone drugs. We report herein the crystal structure of the title compound.

The asymmetric unit of the title compound (Fig. 1) contains one-half molecule, in which a mirror plane passes through S and C4 atoms. The bond lengths (Allen *et al.*,  1987) and angles are within normal ranges.

In the crystal structure, intermolecular N-H···S hydrogen bonds (Table 1) link the molecules (Fig. 2), in which they may be effective in the stabilization of the structure.

### Experimental

For the preparation of the title comppund, 1,2-diaminobenzene (0.019 mol) and water (3 ml) were added to a solution of sodium hydroxide (0.022 mol) in ethanol (20 ml) and carbon disulfide (0.022 mol). The mixture was heated under reflux for 3 h. Charcoal was added cautiously and removed by filtration after the mixture has been refluxed for 10 min more. The filtrate was heated to 377 K and quenched with warm water (377 K, 20 ml), and then acetic acid (9 ml) was added by stirring. The product was separated and after cooling in refrigerator for 3 h the crystallization was completed (Mavrova *et al.*, 2007). Crystals suitable for X-ray analysis were obtained by slow evaporation of an ethanol solution after two weeks.

### Refinement

H atoms were positioned geometrically, with N-H = 0.86 Å (for NH) and C-H = 0.93 Å for aromatic H and constrained to ride on their parent atoms, with U_iso_(H) = 1.2U_eq_(C,N).

### Crystal data

**Table:**

C_7_H_6_N_2_S	*F*(000) = 156
*M**_r_* = 150.21	*D*_x_ = 1.431 Mg m^−^^3^
Monoclinic, *P*2_1_/*m*	Mo *K*α radiation, λ = 0.71073 Å
Hall symbol: -P 2yb	Cell parameters from 25 reflections
*a* = 4.915 (1) Å	θ = 10–14°
*b* = 8.5590 (17) Å	µ = 0.38 mm^−^^1^
*c* = 8.2920 (17) Å	*T* = 294 K
β = 91.76 (3)°	Block, colorless
*V* = 348.66 (12) Å^3^	0.30 × 0.20 × 0.10 mm
*Z* = 2	

### Data collection

**Table:**

Enraf–Nonius CAD-4 diffractometer	647 reflections with *I* > 2σ(*I*)
Radiation source: fine-focus sealed tube	*R*_int_ = 0.044
graphite	θ_max_ = 27.0°, θ_min_ = 2.5°
ω/2θ scans	*h* = 0→6
Absorption correction: ψ scan (North *et al.*, 1968)	*k* = 0→10
*T*_min_ = 0.896, *T*_max_ = 0.963	*l* = −10→10
903 measured reflections	3 standard reflections every 120 min
813 independent reflections	intensity decay: 1%

### Refinement

**Table:**

Refinement on *F*^2^	Primary atom site location: structure-invariant direct methods
Least-squares matrix: full	Secondary atom site location: difference Fourier map
*R*[*F*^2^ > 2σ(*F*^2^)] = 0.049	Hydrogen site location: inferred from neighbouring sites
*wR*(*F*^2^) = 0.152	H-atom parameters constrained
*S* = 1.00	*w* = 1/[σ^2^(*F*_o_^2^) + (0.1*P*)^2^ + 0.059*P*] where *P* = (*F*_o_^2^ + 2*F*_c_^2^)/3
813 reflections	(Δ/σ)_max_ < 0.001
45 parameters	Δρ_max_ = 0.37 e Å^−^^3^
0 restraints	Δρ_min_ = −0.26 e Å^−^^3^

### Special details

**Table:**

Geometry. All e.s.d.'s (except the e.s.d. in the dihedral angle between two l.s. planes) are estimated using the full covariance matrix. The cell e.s.d.'s are taken into account individually in the estimation of e.s.d.'s in distances, angles and torsion angles; correlations between e.s.d.'s in cell parameters are only used when they are defined by crystal symmetry. An approximate (isotropic) treatment of cell e.s.d.'s is used for estimating e.s.d.'s involving l.s. planes.
Refinement. Refinement of *F*^2^ against ALL reflections. The weighted *R*-factor *wR* and goodness of fit *S* are based on *F*^2^, conventional *R*-factors *R* are based on *F*, with *F* set to zero for negative *F*^2^. The threshold expression of *F*^2^ > σ(*F*^2^) is used only for calculating *R*-factors(gt) *etc*. and is not relevant to the choice of reflections for refinement. *R*-factors based on *F*^2^ are statistically about twice as large as those based on *F*, and *R*- factors based on ALL data will be even larger.

### Fractional atomic coordinates and isotropic or equivalent isotropic displacement parameters (Å^2^)

**Table:**

	*x*	*y*	*z*	*U*_iso_*/*U*_eq_	
S	0.06322 (19)	0.2500	0.88609 (10)	0.0510 (3)	
N	−0.2841 (4)	0.1239 (2)	1.1022 (2)	0.0465 (5)	
H0A	−0.2505	0.0281	1.0783	0.056*	
C1	−0.7826 (5)	0.1687 (4)	1.4250 (3)	0.0644 (7)	
H1A	−0.8914	0.1154	1.4964	0.077*	
C2	−0.6229 (5)	0.0844 (3)	1.3201 (3)	0.0561 (7)	
H2A	−0.6243	−0.0242	1.3195	0.067*	
C3	−0.4611 (4)	0.1684 (3)	1.2162 (3)	0.0437 (5)	
C4	−0.1646 (7)	0.2500	1.0292 (4)	0.047	

### Atomic displacement parameters (Å^2^)

**Table:**

	*U*^11^	*U*^22^	*U*^33^	*U*^12^	*U*^13^	*U*^23^
S	0.0671 (6)	0.0270 (5)	0.0588 (6)	0.000	0.0015 (4)	0.000
N	0.0585 (11)	0.0227 (9)	0.0578 (12)	−0.0010 (8)	−0.0061 (9)	0.0009 (8)
C1	0.0621 (14)	0.0558 (17)	0.0755 (18)	−0.0084 (13)	0.0082 (13)	0.0065 (14)
C2	0.0681 (15)	0.0359 (13)	0.0640 (16)	−0.0047 (12)	−0.0032 (13)	0.0047 (11)
C3	0.0484 (11)	0.0302 (12)	0.0519 (13)	0.0009 (9)	−0.0094 (9)	−0.0003 (9)
C4	0.057	0.029	0.054	0.000	−0.019	0.000

### Geometric parameters (Å, °)

**Table:**

S—C4	1.656 (4)	C1—H1A	0.9300
N—C3	1.359 (3)	C2—C3	1.390 (3)
N—C4	1.378 (3)	C2—H2A	0.9300
N—H0A	0.8600	C3—C3^i^	1.398 (4)
C1—C2	1.391 (4)	C4—N^i^	1.378 (3)
C1—C1^i^	1.391 (6)		
			
C3—N—C4	112.1 (2)	C1—C2—H2A	121.2
C3—N—H0A	123.9	N—C3—C2	132.6 (2)
C4—N—H0A	123.9	N—C3—C3^i^	106.27 (12)
C2—C1—C1^i^	121.24 (16)	C2—C3—C3^i^	121.11 (15)
C2—C1—H1A	119.4	N—C4—N^i^	103.2 (3)
C1^i^—C1—H1A	119.4	N—C4—S	128.40 (15)
C3—C2—C1	117.6 (2)	N^i^—C4—S	128.40 (15)
C3—C2—H2A	121.2		
			
C1^i^—C1—C2—C3	0.6 (3)	C1—C2—C3—C3^i^	−0.6 (3)
C4—N—C3—C2	−179.1 (2)	C3—N—C4—N^i^	−1.5 (3)
C4—N—C3—C3^i^	1.0 (2)	C3—N—C4—S	179.5 (2)
C1—C2—C3—N	179.5 (2)		

Symmetry codes: (i) *x*, −*y*+1/2, *z*.

### Hydrogen-bond geometry (Å, °)

**Table:**

*D*—H···*A*	*D*—H	H···*A*	*D*···*A*	*D*—H···*A*
N—H0A···S^ii^	0.86	2.57	3.3798 (19)	158

Symmetry codes: (ii) −*x*, *y*−1/2, −*z*+2.
